# MiR-146a *rs2910164 G > C* polymorphism modulates Notch-1/IL-6 signaling during infection: a possible risk factor for Crohn’s disease

**DOI:** 10.1186/s13099-020-00387-0

**Published:** 2020-10-15

**Authors:** Esra’a Keewan, Saleh A. Naser

**Affiliations:** Division of Molecular Microbiology, Burnett School of Biomedical Sciences, College of Medicine, University of Central Florida, 4110 Libra drive, Orlando, FL 32816 USA

**Keywords:** MicroRNA, MiR-146a, Notch, IL-6, Macrophages, Immune response, Crohn’s, Immunity, COVID-19, Paratuberculosis

## Abstract

**Background:**

MiR-146a, an effector mediator, targets Notch-1 and regulates the innate and adaptive immune systems response. Recently, we reported that Notch-1 signaling plays a key role in macrophage polarization and response during infection. We employed *Mycobacterium avium paratuberculosis* (MAP) infection in Crohn’s disease (CD) as a model to demonstrate the role of Notch-1/IL-6 signaling on MCL-1 based apoptosis and intracellular MAP infection and persistence. This study was designed to investigate the impact of polymorphisms in miR146a on the immune response and infection in our MAP-CD model.

**Methods:**

We determined the incidence of miR-146a *rs2910164 G > C* in 42 blood samples from clinical CD patients and controls. We also measured the effect of *rs291016*4 on expression of Notch-1 and IL-6, and plasma IL-6 protein levels in our study group. Finally, we analyzed the blood samples for MAP DNA and studied any correlation with miR-146a polymorphism. Samples were analyzed for statistical significance using unpaired tow-tailed t-test, unpaired two-tailed z-score and odds ratio. P < 0.05 considered significant.

**Results:**

MiR-146a *rs2910164 GC* was detected at a higher incidence in CD (52.6%) compared to healthy controls (21.7%) *rs2910164 GC* Heterozygous polymorphism upregulated Notch-1 and IL-6, by 0.9 and 1.7-fold, respectively. As expected, MAP infection was detected more in CD samples (63%) compared to healthy controls (9%). Surprisingly, MAP infection was detected at a higher rate in samples with *rs2910164 GC* (67%) compared to samples with normal genotype (33%).

**Conclusions:**

The data clearly associates miR-146a *rs2910164 GC* with an overactive immune response and increases the risk to acquire infection. The study is even more relevant now in our efforts to understand susceptibility to SARS-CoV-2 infection and the development of COVID-19. This study suggests that genetic variations among COVID-19 patients may predict who is at a higher risk of acquiring infection, developing exacerbating symptoms, and possibly death. A high scale study with more clinical samples from different disease groups is planned.

## Introduction

MicroRNAs (miRNAs) are a class of small, noncoding RNA (~ 22 nucleotides in length) that post-transcriptionally regulate one-third of protein-coding genes [1]. They do that by pairing with 3'untranslated regions (UTR) of the targeted messenger RNAs (mRNA), leading to transitional repression and/or cleavage of mRNA [2]. MiRNAs also regulate numerous cellular processes, including development, proliferation, differentiation, and metabolism [3, 4]. Recently, miRNAs have been described as a novel mediator of the host immune response against infection, mostly through regulating proteins involved in innate and adaptive immune systems [5].

MiR-146a, a member of miRNAs family, encoded on human chromosome 5q33.3, has an essential role in the regulation of immune cell differentiation, inflammatory cytokines production, host defense, and various immunological conditions [6, 7]. Interestingly, recent studies reported an important role of miR-146a in macrophages polarization [8, 9]. For example, Hung et al. reported that miR-146a could diminish pro-inflammatory M1 macrophages response by promoting M2 polarization [8]. MiR-146a seems to have protective effect in inflammatory conditions by negatively regulating pro-inflammatory cytokines production, thus avoiding the expansion of harmful inflammatory responses and preserve the homeostatic status [10]. An association between aberrant miR-146a expression and various pathological settings has been consistently reported [11]. A dysregulated expression of miR-146a and their targets have been associated with a functional polymorphism in miR-146a sequence [12]. Various studies reported that *rs2910164 GC* (single nucleotide polymorphism) on the passenger strand of miR-146a precursor has been associated with many pathological conditions including Rheumatoid Arthritis (RA) [13], severe sepsis [14], tuberculosis [15], and cancer[16]. Specifically, *rs2910164 GC* polymorphism in Mir146a- has been shown to cause reduction in mature miR-146a expression level and the silencing of its target genes by interfering with the processing of pre-miRNA [12].

Notch signaling is a highly conserved juxtracrine signaling, which has been identified as a critical regulator of immune cell development and functions [17]. Recently, we reported the involvement of Notch-1 signaling in macrophages immune response and defense mechanisms against *Mycobacterium avium paratuberculosis* (MAP) infection. Specifically, our study demonstrated that MAP could modulate macrophage responses which led to its survival, higher bacterial load, and ultimately chronic persistence of the infection [18]. Most importantly, we linked macrophage modulation with the induction of Notch-1 and IL-6 signaling and its downstream influence on myeloid cell leukemia sequence-1 (MCL-1), which results in delayed apoptosis, and severe inflammation [18].

MAP has been associated with several autoimmune diseases including Crohn’s disease (CD), RA, and others [19–21]. Our lab has been investigating CD for decades and significant progress has been made toward disease etiology, diagnostics, and treatment [21–23]. Briefly, CD is a chronic, relapsing disorder of gastrointestinal tract that is pathologically characterized by intestinal inflammation and transmural epithelial injuries [24]. The incidence and prevalence of CD have been increased markedly over the past decade [25]. Recently, Inflammatory bowel disease (IBD) including CD has been stated as the second most common chronic inflammatory disease after RA [26]. CD typically presents with potentially debilitating symptoms which could adversely affect the patient’s quality of life, ability to work, and overall well-being [24, 27]. It is believed that CD is a consequent of a complex interaction between multi-factors including, environmental, genetics, microbial, and host immune system [24, 26]. The combination of these factors seems to induce deterioration in intestinal homeostasis, therefore, allowing penetration of luminal antigens into the bowel wall, resulting in uncontrolled inflammatory responses of the gut in a genetically susceptible host. MAP is considered one of the most extensively studied pathogens associated with CD pathogenesis [21, 28]. Although MAP has been detected in most CD patients [21], the presence of MAP in healthy individuals supports the hypothesis that host genetics and environmental stress may contribute to disturbing the immune responses against MAP infection that lead to the development of CD. This hypothesis is significantly supported by Genome-wide association studies which reported an overlap of genetic susceptibility to CD with susceptibility to MAP infection. Of particular interest, SNPs in genes that are important in anti-bacterial responses of the innate and adaptive immunity, such as Nucleotide-binding oligomerization domain-containing protein 2 (NOD2) and PTPN2/22 have been consistently reported in CD [22, 29].

MAP survival in macrophages depends significantly on Notch-1 signaling [18]. Interestingly, Notch-1 was identified as a potential target gene of miR-146a [8, 30]. He et al. reported that the miR-146a overexpression significantly reduced Notch-1 and subsequently decreased IL-6 production in LPS activated RAW264.7 macrophages [30]. In this study, we investigated the possible interaction between Notch-1 and miR-146a polymorphism which may interfere with Notch-1 expression and downstream signaling and susceptibility to MAP infection in CD. To the best of our knowledge, no study investigated this interaction. We hypothesized in the current study that macrophages from patients with SNP in miR-146a may have higher Notch-1 expression and increase the incidence of MAP infection. Specifically, this study focused on investigating the prevalence of miR-146a *rs2910164* SNP in blood from CD patients in association with MAP infection and expression of Notch-1 and IL-6.

## Results

### Genotype and allele frequency distributions among CD patients and healthy controls

**Fig. 1:**
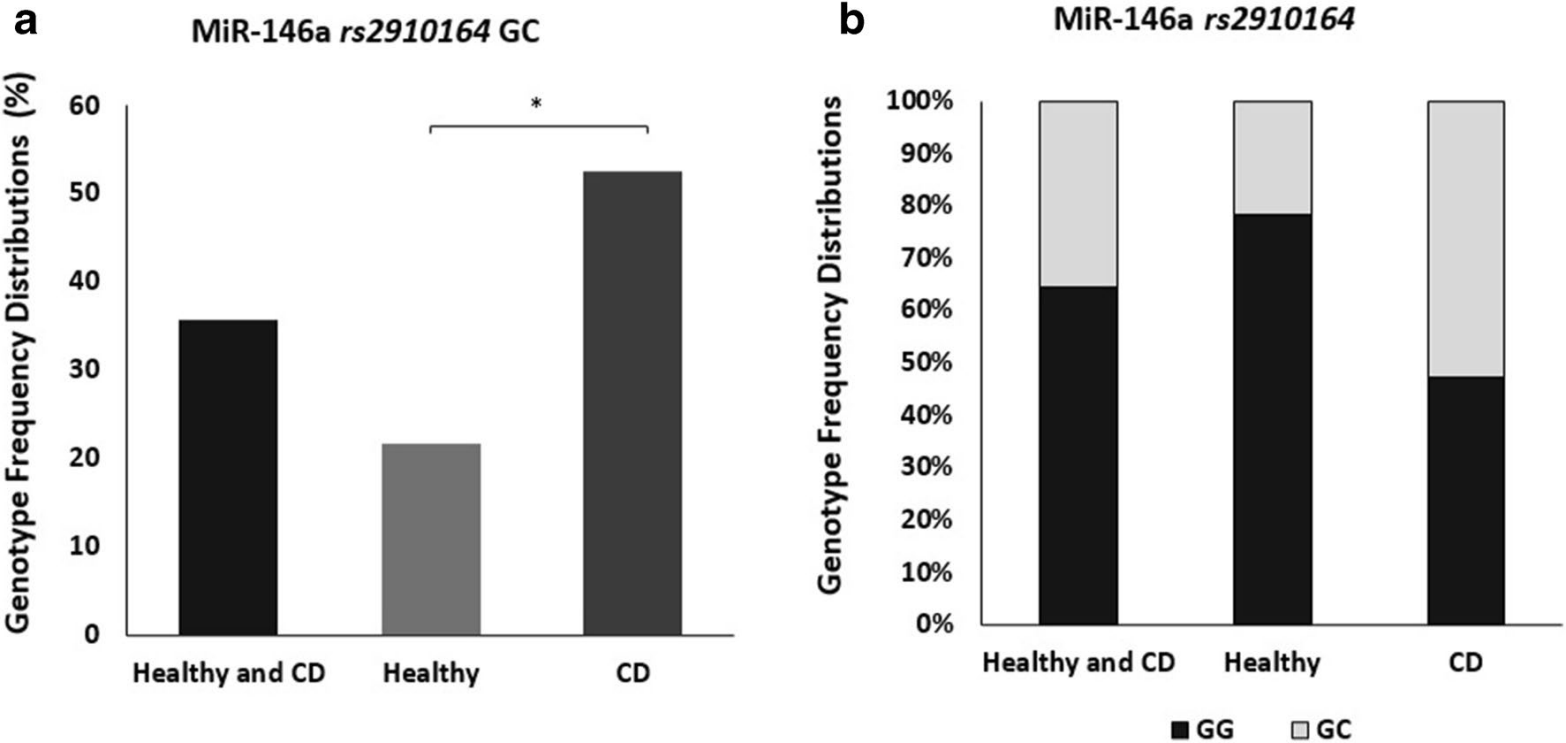
Genotype and Allele frequency distributions among CD patients and healthy controls. **a** MiR-146a *rs2910164 GC* genotype frequencies. **b** MiR-146a *rs2910164* genotype frequencies. G: major allele and C: minor allele. Data presented as percentages. *P < 0.05

Allelic, genotyping frequencies for miR-146a *rs2910164* SNP were determined in 42 subjects (19 CD patients and 23 healthy controls) using TaqMan^™^ genotyping assay. The distributions of the genotype frequencies for the SNP in both CD patients and healthy controls fit Hardy-Weinberg equilibrium. MiR-146a *rs2910164* homozygous major allele (GG) is considered normal/no SNP, while heterozygous allele (GC) and homozygous minor allele (CC) were considered abnormal genotypes and defined as SNP positive. Initially, we determined the occurrence of *rs2910164* amongst all study participants regardless of disease status (CD and healthy control). As shown in Fig. 1a, b, *rs2910164* heterozygous genotype GC occurred at (35.7%) in all samples, However, the minor homozygous CC genotype was not detected in any participant. The heterozygous GC genotype was detected significantly higher in CD patients at 52.6% compared to 21.7% in healthy controls (P < 0.05, OR = 4.0; 95% CI 1.05–15.26).

### MiR-146a *rs2910164 GC* is associated with MAP infection

**Fig. 2:**
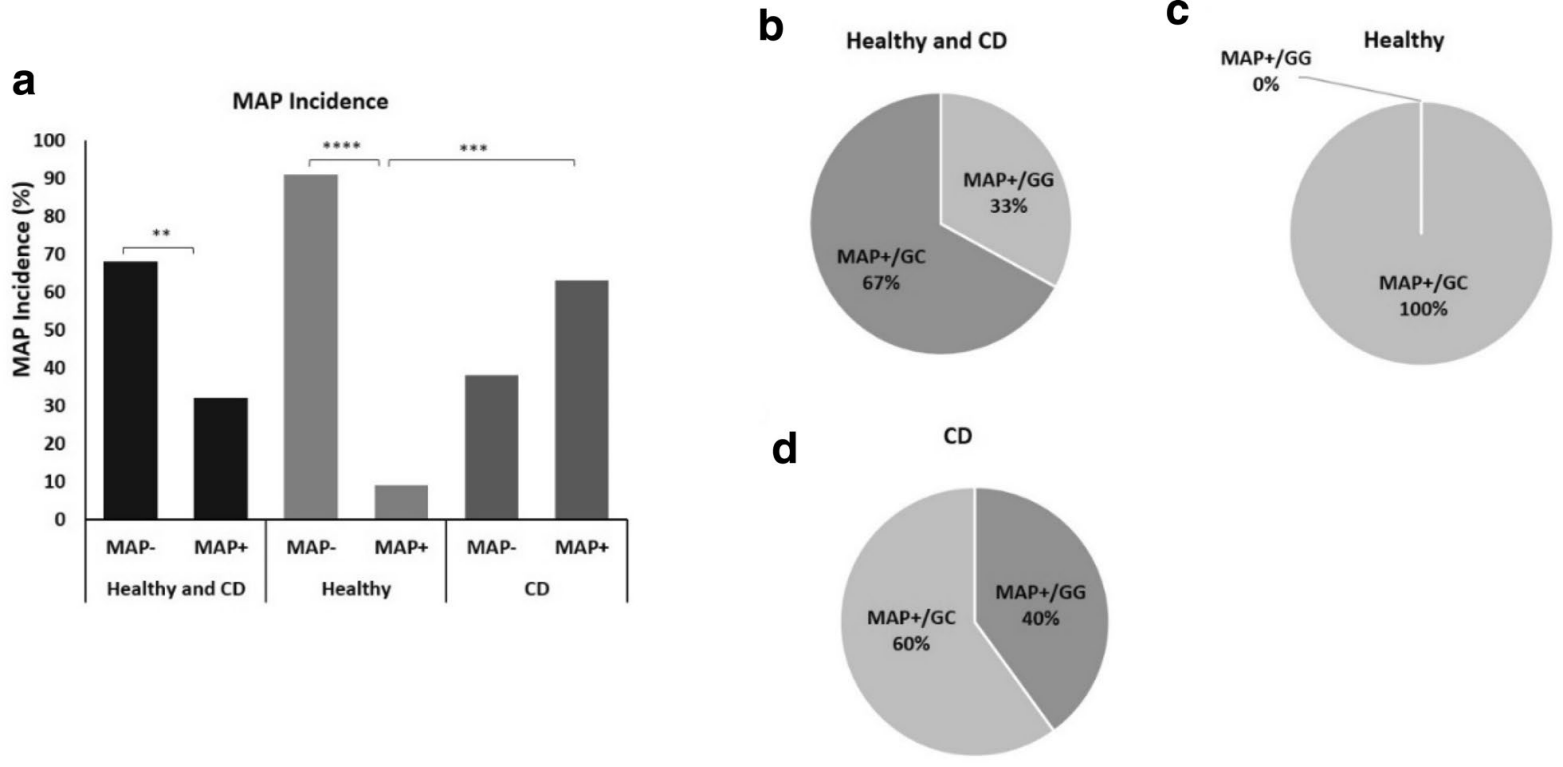
*Mycobacterium avium paratuberculosis* (MAP) infection incidence and association with miR-146a *rs2910164 GC* genotype in CD and healthy controls. **a** The presence of MAP DNA in blood samples from CD and healthy controls was examined by nPCR. **b**–**d** MAP incidence according to *rs2910164* genotypes. Data presented as percentages. **P < 0.01, ***P < 0.001, ****P < 0.0001

To explore the effect of *rs2910164 GC* in mir146a on MAP infection susceptibility in CD patients and healthy controls, we used nPCR to determine the presence of MAP DNA in Buffy coat blood samples obtained from all consented participants. As shown in Fig. 2a, MAP DNA was detected in 32% of all study participants, regardless of disease status. Interestingly, 63% of CD patients were MAP positive compared with only 9% of healthy controls (P < 0.001, OR = 16.7; 95% CI 2.8–97.96).

Then we performed correlation analyses to examine a possible association of *rs2910164 GC* genotype with MAP infection in clinical samples. Surprisingly, MAP DNA was detected in 67% of samples with *rs2910164 GC* genotype, regardless of disease status (OR = 4.0; 95% CI 0.73–21.8; Fig. 2b). In healthy control samples, MAP DNA was detected only in samples with *rs2910164 GC* genotype (Fig. 2c). In CD patients, 60% of MAP positive incidence was observed in *rs2910164 GC* genotype (OR = 2.25; 95% CI 0.38–13.5; Fig. 2d).

### Expression and protein levels of Notch-1/IL-6 in blood from Crohn’s disease

**Fig. 3:**
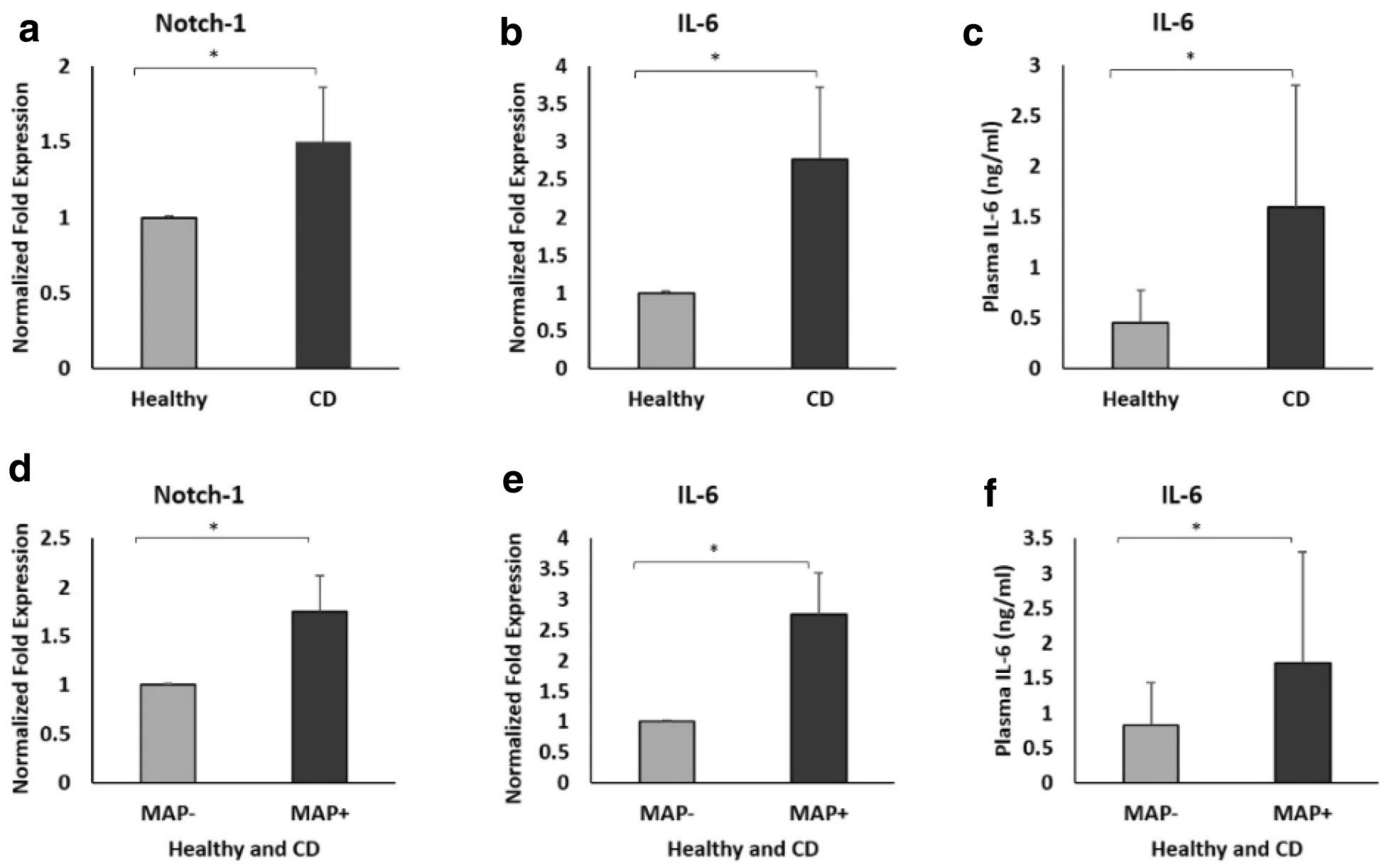
Upregulation of Notch-1 and IL-6 in the blood of CD patients and the effect of *Mycobacterium avium paratuberculosis* (MAP) infection on Notch-1 and IL-6 levels. **a**, **b** Expression levels of Notch-1 and IL-6 were measured in peripheral leukocytes isolated from CD and healthy controls (n = 4). **c** Circulating IL-6 levels were measured in the plasma of CD and healthy controls (n = 22). **d**, **e** Expression levels of Notch-1 and IL-6 were measured in peripheral leukocytes isolated from MAP positive and MAP negative individuals (n = 4). **f** Circulating IL-6 levels were measured in the plasma of MAP positive and MAP negative individuals (n = 22). Gene expression levels were measured using RT-PCR. Plasma IL-6 levels were measured using ELISA. Data are shown as mean ± SD, and significant is considered as *P < 0.05

To further elucidate the possible involvement of Notch-1 and IL-6 in the susceptibility of MAP infection and CD pathogenesis, Notch-1 and IL-6 gene expression levels were measured in purified leukocytes from CD patients and healthy controls. Furthermore, circulating IL-6 levels were measured in plasma of all samples used in this study. As shown in Fig. 3a, b, the overall expression of both Notch-1 and IL-6 gene was significantly upregulated by 0.5 and 1.8-fold, respectively in CD compared to healthy control (P < 0.05). Likewise, plasma IL-6 level was significantly higher in CD patients (1.6 ± 1.2 ng/ml) compared to healthy control (0.45 ± 0.32 ng/ml) (P < 0.05; Fig. 3c). We further determined possible association between MAP infection and Notch-1 and IL-6 levels. As shown in Fig. 3 d, e, the expression of Notch-1 and IL-6 were 0.75 and 1.75-fold, respectively higher in MAP positive subjects compared to MAP negative subjects (P < 0.05).

At the protein level, circulating IL-6 levels were also measured in plasma from participants subjects. IL-6 level was significantly higher in the plasma of MAP positive subjects (1.72 ± 1.65 ng/ml) compared to samples from MAP negative subjects (0.82 ± 0.74 ng/ml) (P < 0.05; Fig. 3f).

### Influence of the miR-146a *rs2910164* genotypes on the expression and Protein levels of Notch-1/IL-6

**Fig. 4:**
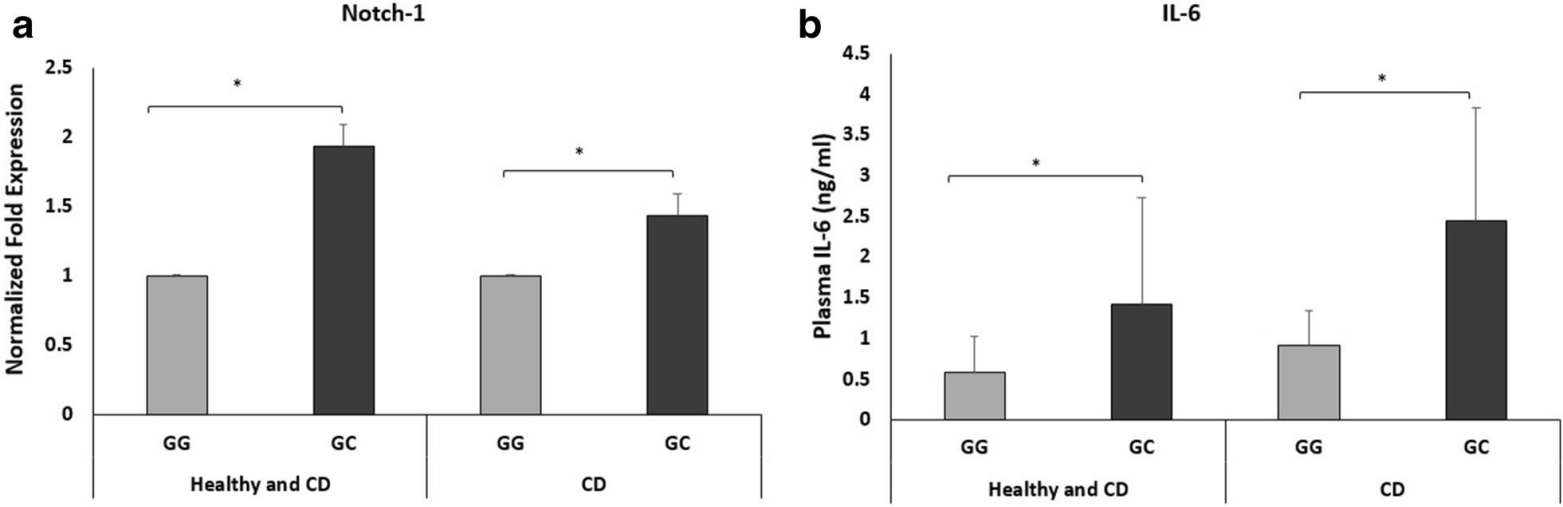
Notch-1 and IL-6 levels in the blood of CD patients and healthy controls according to *rs2910164* miR-146a genotypes. **a** Expression level of Notch-1 was measured in peripheral leukocytes isolated from CD and healthy controls (n = 4). **b** Circulating IL-6 levels were measures in the plasma of CD and healthy controls (n = 22). Notch-1 gene expression was measured using RT-PCR. Plasma IL-6 levels were measured using ELISA. Data are shown as mean ± SD, and significant is considered as *P < 0.05

To investigate whether polymorphism in miR-146a affect expression and function of potential target genes, we examined the expression levels of Notch-1 in peripheral leukocytes separated from the blood of CD patients and healthy controls. Also, we measured circulating IL-6 protein levelin the plasma samples of both groups. As shown in Fig. 4a, Notch-1 gene expression was upregulated in *rs2910164 GC* genotype by 0.94-fold compared to wildtype genotype regardless of disease status (P < 0.05). In CD, Notch-1 was significantly upregulated among *rs2910164 GC* genotype carriers by 0.44-fold compared to wildtype carriers (P < 0.05). Likewise, at the protein level, circulating IL-6 levels were significantly upregulated in subjects with *rs2910164* who had GC genotype (1.42 ± 1.31 ng/ml) compared with normal subjects (0.6 ± 0.45 ng/ml) regardless of disease status (Fig. 3b; P < 0.05). In CD, plasma IL-6 levels were significantly upregulated in subjects with *rs2910164 GC* genotype (2.45 ± 1.4 ng/ml) compared to normal subject (0.91 ± 0.05 ng/ml) (Fig. 4b; P < 0.05).

## Discussion

The pathogenesis of CD involves a complex interaction between environmental, genetics, microbial, and dysregulated immune response [24, 26]. Currently, the most accepted pathogenic model of CD is based on an inefficient response of the immune system to intestinal microbial triggers in genetically susceptible individuals [31]. MAP has long been considered a potential microbial trigger in the pathogenesis of CD and other autoimmune disorders [19–21]. Thus, identification of the molecular and genetic basis for susceptibility and immune response against MAP infection would facilitate the development of effective and preventative interventions to combat MAP infection in humans. Recently, we reported novel mechanistic findings that are key in the involvement of Notch-1 signaling and its downstream effect on IL-6 and MCL-1 expression in infected macrophages. Specifically, MAP infection in macrophages induced Notch-1 and IL-6 signaling and resulted in the hijacking of MCL-1 causing apoptosis and persistence infection. MAP-Notch-1 interaction modulated macrophage polarization toward M1 pro-inflammatory phenotypic response [18]. The study clearly supported the role of Notch-1 signaling in MAP infection in autoimmune diseases. This finding intrigued us to investigate other factors that may be involved in MAP-Notch-1 interaction such as miRNA.

MiRNAs are known to play essential roles in the pathogenesis and control of infectious disease [5, 7]. They regulate genes involved in differentiation, functions, and modulation of immune response [5, 7]. MiR-146a has been identified as an effector mediator to regulate the magnitude of the inflammatory response and tissue damage due to their direct influence on immune cell differentiation and cytokines production [8, 10, 30]. Alterations in miR-146a expression due to SNP may lead to loss of function and may reverse the beneficial effect expected from miR-146a during infection. We became intrigued by *rs2910164* which includes G > C nucleotide replacement in the stem structure of pre-miR-146a for possible involvement in MAP-Notch-1 interaction. We designed this study to investigate the incidence of *rs2910164* in clinical samples from CD patients. We were surprised to observe that it occurred in 53% in CD compared to 22% in healthy controls. Although we didn’t detect any homozygous minor allele genotyping CC in any samples, we were surprised to observe upregulation in Notch-1 and IL-6 expression due to the GC heterozygous allele which was 0.9 and 1.7-fold upregulation compared to normal genotype samples. This loss of inhibitory function of miR146a may have an impact on modulation of macrophage immune response and infection. This led us to examine any possible correlation between *rs2910164 GC* and susceptibility to MAP infection in CD.

To the best of our knowledge, the present study was the first to examine the prevalence of miRNA-146a gene polymorphism rs2910164 in CD in relation to Notch-1 signaling and MAP infection. Our data demonstrated a significant prevalence of *rs2910164 GC* genotype in CD compared with healthy controls. The rs2910164 GC genotype was associated with loss of mir146a function and an increase in Notch-1 and IL-6 expression. These findings may explain why *rs2910164* is prevalent in inflammatory diseases and it suggests that it may be considered a susceptibility factor for pro-inflammatory response and infection in these patients. Our results demonstrated a high incidence of MAP infection in CD samples compared with healthy controls. Specifically, we analyzed the association of *rs2910164 GC* with MAP infection, and we found that the majority of MAP infection was among *rs2910164 GC* carriers either CD or healthy controls. Particularly, in healthy controls, 100% of MAP infection was among *rs2910164 GC* carriers. This finding strongly supports the involvement of *rs2910164 GC* in host immune response and susceptibility to MAP infection. The association of *rs2910164 GC* with infection was reported earlier by Li et al., who linked *rs2910164 GC* with *M. tuberculosis* infection [15]. However, the molecular basis of such involvement was never reported before the study described here.

Given the critical role of Notch-1 signaling on modulating macrophages immune response against MAP infection [18]. First, we investigated the expression levels of Notch-1 and IL-6 in the blood of CD and healthy controls. Our data indicated significant upregulation of Notch-1 and IL-6 gene expression in the peripheral leukocytes isolated from CD compared with healthy controls. Consistently, our results also demonstrated significant upregulation of circulating IL-6 in the plasma of CD compared with healthy controls. Elevated IL-6 in active CD was reported earlier by other studies [32]. Then to elucidate the association of MAP infection with Notch-1 and IL-6 levels, we analyzed Notch-1 and IL-6 levels in MAP positive versus MAP negative samples. Our results indicated upregulation of Notch-1 and IL-6 levels in MAP positive samples compared with MAP negative samples. These findings are strongly consistent with our previous in vitro study that MAP induce Notch-1 signaling in infected macrophages [18]. Particularly, our previous study reported interplay between Notch-1 and IL-6 expression in vitro, we provided evidence that Notch-1 signaling induces IL-6 expression in macrophages, which in turn induces Notch-1 signaling activation to start feed-forward loop to amplify the immune response [18].

Finally, to identify the influence of *rs2910164 GC* on Notch-1 and IL-6 levels in CD. We investigated the expression levels of Notch-1 in peripheral leukocytes and circulating IL-6 among *rs2910164* genotypes. We were surprised to see strong and significant correlation between *rs2910164 GC* genotype and increase in Notch-1 expression and IL-6 cytokine production. This is a new finding that strongly identifies miR-146a *rs2910164 GC* as functional SNP that modulate immune response against intracellular infection. The finding clearly associates *rs2910164 GC* genotypes with loss of function in miR-146a, and therefore, exerts contradictory regulatory effects on Notch-1 and IL-6 expression. Consequently, this leads to uncontrolled inflammatory response and potential tissue damage. This may explain the excessive increase in IL-6 level in conditions associated with Crohn’s disease, RA, Tuberculosis, and most recently in SARS-CoV-2 [32–35]. In fact, high IL-6 level has been identified as a predictor of COVID-19 mortality [34], it was reported recently that IL-6 trans-signaling may be responsible for infiltration of granulocytes and monocytes and dame in lung tissue in cases with SARS-CoV-2 infection [36]. We propose that miR-146a *rs2910164 GC* through Notch-1 upregulation causes excessive secretion of IL-6 which may then affect cellular uptake of SARS-CoV-2, development of COVID-19, and contribute to the cytokine storm in patients. The occurrence of miR-146a *rs2910164* in the majority of CD patients may increase their susceptibility to SARS-CoV-2 infection. This possibly could increase commodities among patients with underlying conditions and increases mortality during the COVID-19 Pandemic. The findings in this exploratory study should intrigue other investigators to pursue our observations. Certainly, our group plans to seek collaboration with clinicians to access clinical samples from CD and RA patients who were exposed or infected with SARS-CoV-2. Specifically, weplan to study Clinical samples from COVID-19 patients with comorbidities for the presence of *rs2910164 GC* and effect on IL-6 level.

**Fig. 5:**
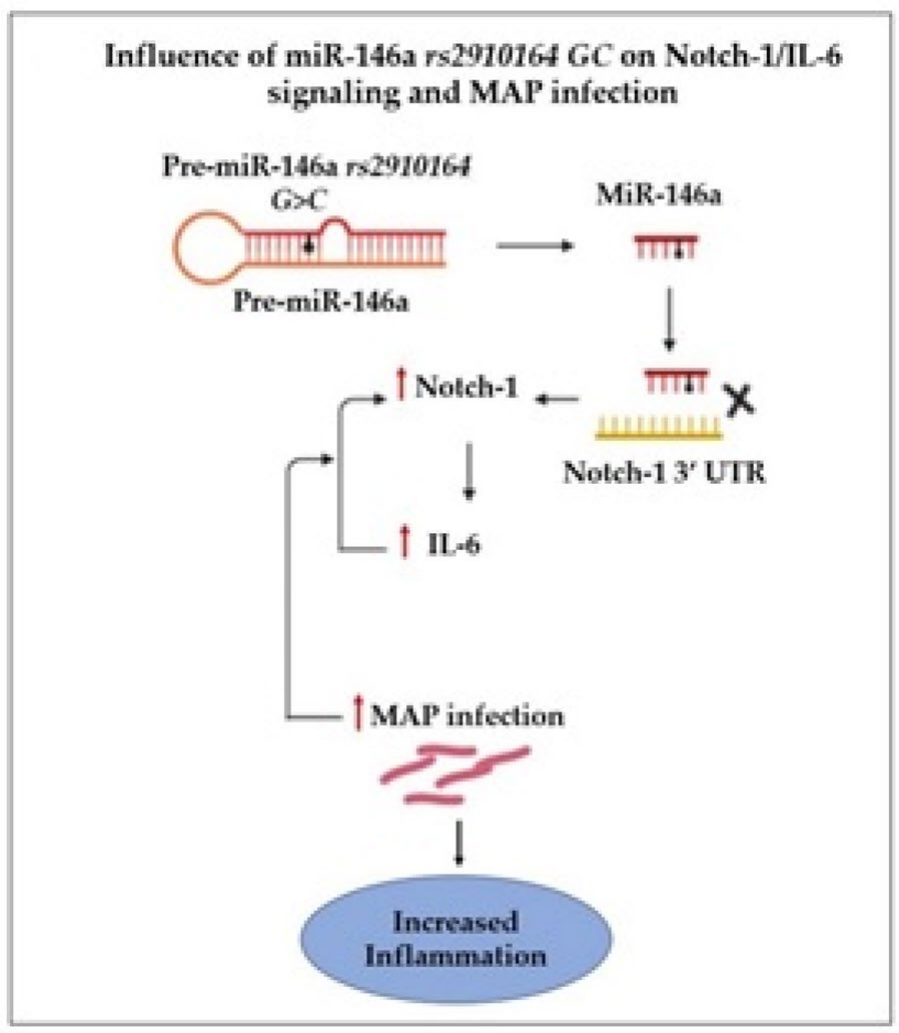
Schematic illustration of the study. *rs2910164* includes G > C replacement in the stem structure of pre-miR-146a which led to loss of anti-inflammatory effect on Notch-1 and IL-6. This caused upregulation in Notch-1/IL-6 and enhanced MAP infection and inflammation

In conclusion, the data clearly associates miR-146a *rs2910164 GC* with an overactive immune response and increases the risk to acquire infection (Fig. 5). The study is even more relevant now in our efforts to understand susceptibility to SARS-CoV-2 infection and the development of COVID-19. Genetic variations among COVID-19 patients may predict who is at a higher risk of developing exacerbating symptoms, severe illness and possibly death. The recent promising outcome of anti-IL-6 monoclonal antibodies therapy in COVID-19 patients supports the conclusion of this study that Notch-1/IL-6 signaling is key to understand and treat inflammation[37].

## Materials and methods

### Clinical samples and processing

**Fig. 6:**
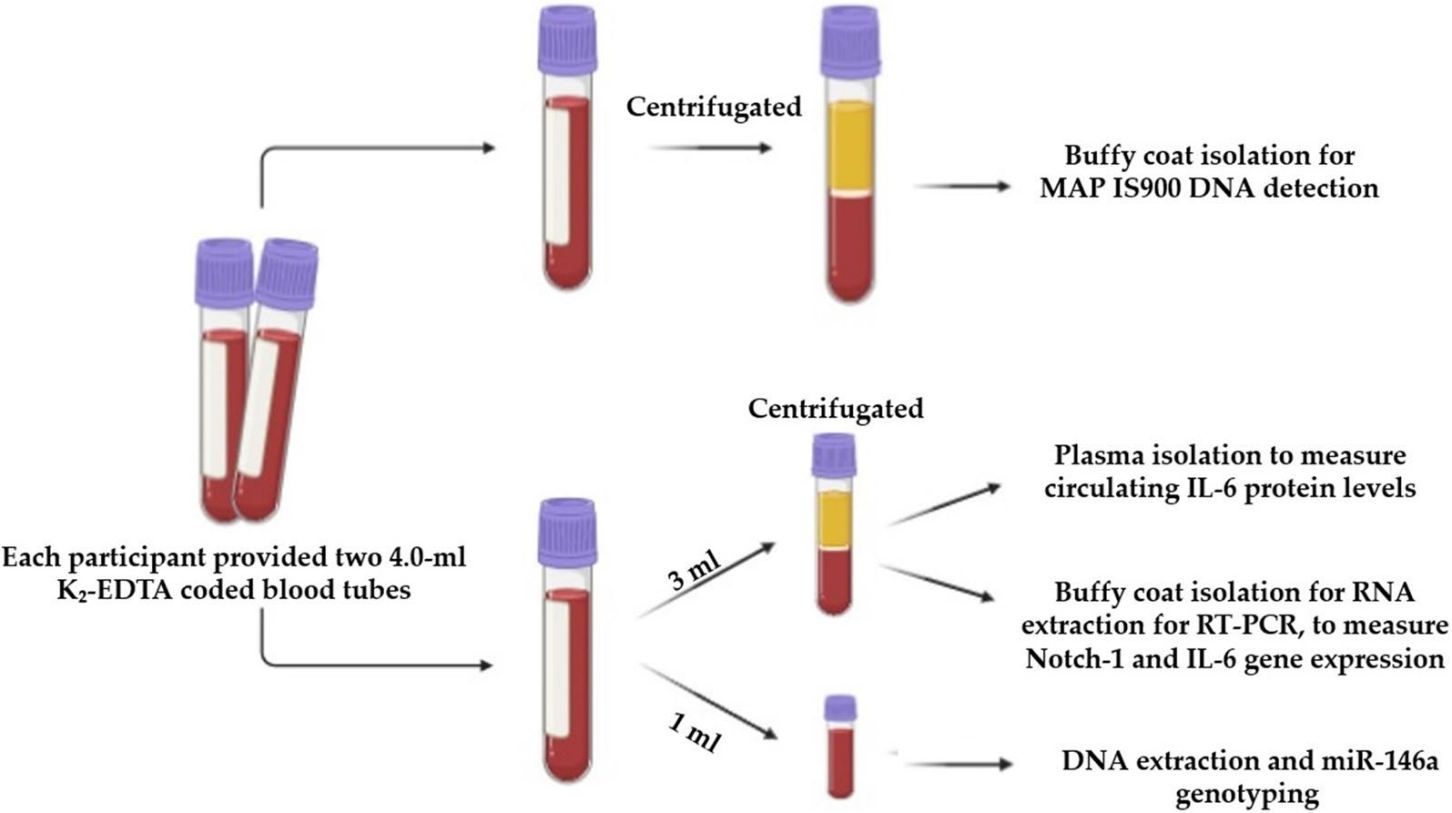
Clinical samples collection and processing procedure used in this study

Peripheral blood (two 4.0-ml K2-EDTA coded tubes) were collected from 42 subjects (19 CD patients and 23 healthy controls). All participants completed and signed a written informed consent prior to enrolment in the IRB approved study #IRB00001138. The average age and male/female ratio of all participants with CD and healthy controls are in Table 1. Although the samples included in this study originated from an ongoing blinded study, for sure we know that the CD samples were obtained from patients who were well diagnosed with approved standard diagnostic guidelines for moderate to severe CD symptoms. The first blood tube was processed for Buffy coat isolation and used for DNA extraction and nPCR testing for presence of MAP DNA. The second blood tube from each participant was aliquoted into different tubes where some used for DNA extraction for *rs2910164* miR-146a genotyping, others were subjected to RNA extraction for RT-PCR analysis of Notch-1 and IL-6 expression, and the remainder plasma was used to measure circulating IL-6 protein level, Fig. 6.

**Table 1:** Demographics of study participants

Diagnosis	Number	Average age (range) year	Male:female (%)
All subjects	42	36 (21–66)	44:56
Crohn’s disease	19	42 (25–66)	56:44
Healthy	23	31 (21–62)	33:67

### Extraction of DNA and detection of MAP IS900 DNA in peripheral leukocytes

Blood sample tube designated for MAP DNA detection was centrifuged at 3000 RPMs, and a buffy coat layer containing peripheral leukocytes was transferred to a new sterile 1.5 ml microcentrifuge tube. After 10 min incubation with RBC lysis buffer (G-Biosciences®, Cat# 786 − 649, MO, USA), the tube was centrifuged at 5000 RPMs for 5 min and then the purified buffy coat pellet was stored in Tris-EDTA (TE) buffer at − 20 °C until further use.

DNA extraction was performed using a modified DNAzol® extraction protocol as described earlier [19]. MAP IS900 DNA detection was done following our nested PCR (nPCR) protocol as described earlier [20]. Briefly, nPCR protocol consists of two amplification rounds and based on MAP-specific IS900 derived oligonucleotide primers. First-round (95 °C for 5 min, then 34 cycles of 95 °C for 1 min, 58 °C for 1.5 min, 72 °C for 1.5 min. Final extension of 10 min at 72 °C) was performed using P90 (5′-GTTCGGGGCCGTCGCTTAGG-3′) and P91 (5′-GAGGTCGATCGCCCACGTGA-3′) primers (Eurofins, KY, USA), which produce a 398 bp amplicon. The second round of amplification was completed using AV1 (5′-ATGTGGTTGCTGTGTTGGATGG-3′) and AV2 (5′-CCGCCGCAATCAACTCCAG-3′) primers (Eurofins, KY, USA) with a final amplification of 298 bp amplicon. The second round protocol included 95 °C for 5 min, then 34 cycles of 95 °C for 1 min, 60 °C for 0.5 min, 72 °C for 1.5 min and a final extension step of 10 min at 72 °C. Then the PCR final products from the second round were analyzed on 2% agarose gel.

## MiR-146a genotyping

Genomic DNA was purified from peripheral blood using QIAamp® DNA Blood Mini Kit (Qiagen™, Cat# 51,104, MD, USA) following manufacturer’s protocol. Genotyping of miR-146a for *rs2910164* SNPs was performed at the University of Florida pharmacotherapy and Translational Research Department (Gainesville, FL) using TagMan^™^ SNP Genotyping Assay (Applied Biosystems™, CA, USA) as described previously [19]. Briefly, reaction mixtures composed of 2× TaqMan™ Master Mix and 20x Assay Working Stock (primers with VIC and FAM fluorophore attachment) were loaded into a 384-well microtiter plate. Then isolated DNA and controls were added to the plate which then was subjected to RT-PCR (95 °C for 10 min for 1 cycle, 92 °C for 15 s and 58 °C for 1 min for 50 cycles) using Applied Biosystems™ QuantStudio™ RT-PCR System. The plate was analyzed for VIC and FAM fluorophores for each sample at 551 and 517 nm, respectively. Fluorescence of VIC or FAM alone determined that the sample had allele 1 or allele 2, while VIC and FAM together determined that the sample is heterozygous for each allele.

## Measurement of gene expression by quantitative real-time PCR (RT-PCR)

RNA was isolated from peripheral leukocytes using TRIzol™ reagent (Thermo Fisher, Cat# 15,596,018) according to the manufacturer’s instructions and then was used to synthesize cDNA. Notch-1 and IL-6 gene expression was measured using StepOnePlus^™^ Real-Time PCR System (Thermo Fisher, Cat# 4,376,600), Fast SYBR Green Mastermix (Thermo Fisher, Cat# 4,385,610) was used as a detection dye. Housekeeping β-actin primers (forward primer: 5′-CTCATCTTGTTTTCTGCGCAAGTT-3′; reverse primer: 5′-CTTCCCTCCTCAGATCATTGCTC-3′) (Thermo Fisher) was used to measure the endogenous baseline CT values. Relative mRNA expression levels were calculated by using the Eq. 2(−ΔCT) × 1000, where ΔCT= [(Sample RT-PCR CT value) − (β-actin CT baseline value)]. Notch-1 primers (forward primer: 5′-TGAAATTCAGGGCCCCTCC-3′; reverse primer: 5′-GCATCGGGCACCTGAAC-3′), IL-6 primers (forward primer: 5′-AGGAGAAGATTCCAAAGATGTAGCC-3′; reverse primer: 5′-TGCTCTAGAACCCAGCAAAGAC-3′).

### Measurement of circulating IL-6 levels by enzyme-linked immunosorbent assay (ELISA)

Circulating IL-6 levels were measured in the plasma using IL-6 Human ELISA kit (Thermo Fisher, Cat# BMS213HS) following the manufacturer’s instructions. IL-6 levels were determined by reading optical density at 450 nm using SpectraMAX® i3x Multi-mode microplate reader.

### Statistical analysis

Samples were analyzed for statistical significance using unpaired tow-tailed t-test, unpaired two-tailed z-score and odds ratio. All statistical analyses were performed using Prism 8 (GraphPad software, version 8.4.3, San Diego, CA, USA). P < 0.05 considered significant.

## Data Availability

The datasets supporting the conclusions of this article are included within the article.
